# Proximal Femoral Nail Mechanical Failure: A Case Report and Biomechanical Study

**DOI:** 10.7759/cureus.23694

**Published:** 2022-03-31

**Authors:** Dimitrios Papanikolopoulos, Christos Kalligeros, Apostolos Polyzos, Vasileios Spitas, Vasileios Soranoglou

**Affiliations:** 1 2nd Department of Orthopaedics and Traumatology, Athens General Hospital "G. Gennimatas", Athens, GRC; 2 Laboratory of Machine Design, National Technical University of Athens, Athens, GRC

**Keywords:** implant breakage, fractography, pertrochanteric fracture, mechanical failure, fixation failure

## Abstract

Intramedullary nailing is an established method for treating pertrochanteric fractures. However, the widespread use of this technique comes along with a variety of complications. We present a case of a 50-year-old female who presented to the emergency department suffering a left pertrochanteric fracture. She was treated with proximal femoral nailing and discharged home. Nine months later, she presented again to the emergency department with pain and an inability to bear weight. Imaging revealed the mechanical failure of the hip screw and loss of fracture fixation. Revision surgery included extraction of the broken hardware and a left hip hemiarthroplasty. The removed implant was sent for further evaluation.

Fractographic analysis showed acute breakage due to bending and torsion forces acting on the screw with no relevant signs of metal fatigue. This biomechanical method is of great value for the surgeon and the implant manufacturer in order to understand the failure pattern and optimize future implants and fixation techniques. Improved implant biomechanical properties together with meticulous surgical technique constitute the cornerstones for optimal results.

## Introduction

Intramedullary nailing is used for more than 25 years in the treatment of stable and unstable pertrochanteric fractures [1-3]. Due to the continuous increase in the number of proximal femoral fractures and relevant surgeries, complications such as loss of fixation, peri-implant femoral fracture, osteonecrosis, infection, and nonunion [4,5] rise as well. Mechanical failure of proximal femoral nails is rare but can be disastrous. The salvage surgical procedures that are required in these cases are very demanding and have a severe impact on the patient’s health [6]. Breakage of nails has been previously studied in the literature and several patterns of fixation failures and implant breakage have been reported [6-13]. Moreover, biomechanical studies have documented weak points in nail design and suggested methods to optimize nail mechanical performance [6,12-16]. In this paper, we present a case report of a patient who suffered a proximal femoral nail breakage in an unusual pattern.

## Case presentation

**Figure 1:**
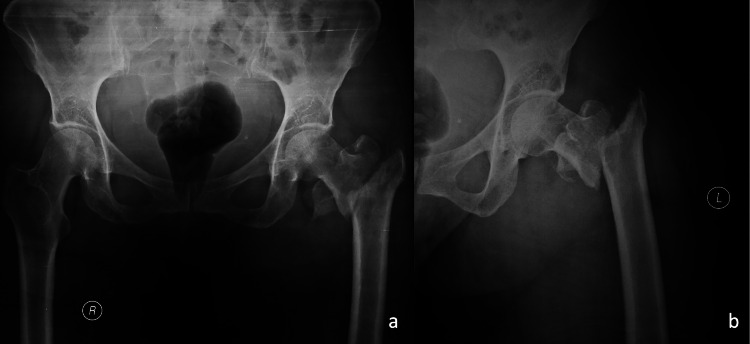
Anteroposterior pelvis (a) and lateral left hip (b) x-rays.

**Figure 2:**
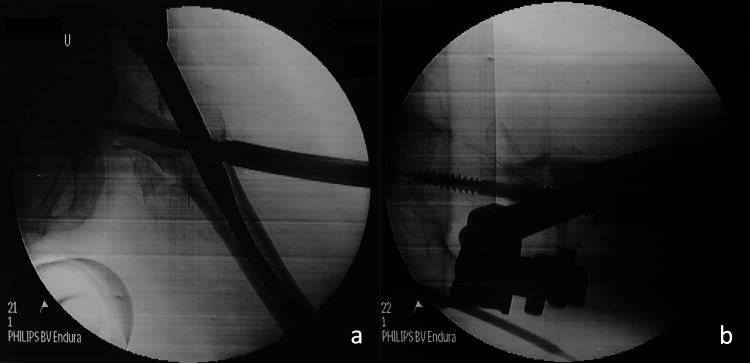
Anteroposterior left hip (a) and lateral left hip (b) intra-op fluoroscopy.

A 50-year-old female with a body mass index (BMI) of 19,5 was transferred to the Emergency Department after a fall from a standing height. She sustained a displaced pertrochanteric fracture (AO A2-2B) of her left hip (Figures 1a, 1b). Her past medical history was significant for hypertension and thyroid dysfunction. On post-admission day three, the patient underwent surgical fixation of her left hip on a fracture table. A proximal femoral nail (KFN, Königsee Implantate, Allendorf, Germany) of 125^ο^ Caput-Collum-Diaphyseal (CCD) angle was implanted. A hip screw of 80 mm length was used and distal locking was achieved by means of a 34-mm distal screw-in dynamic mode (Figures 2a, 2b). Postoperatively, the patient was mobilized with weight-bearing as tolerated and was discharged from the hospital on postoperative day 4. Screening for osteoporosis and vitamin D levels was scheduled but she was lost to follow-up.

**Figure 3:**
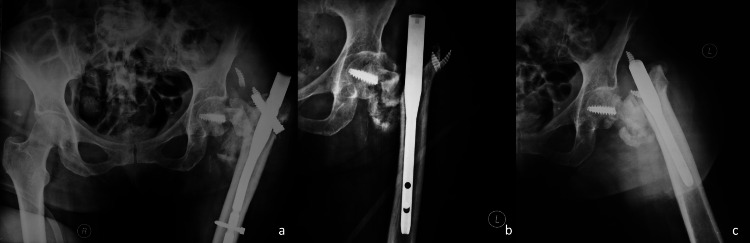
Anteroposterior pelvis (a) and lateral left hip (b,c) x-rays after implant failure.

Nine months after the primary operation, the patient was transferred again to the emergency department, reporting pain and inability to bear weight on her left hip without reporting clearly what happened. X-rays (Figures 3a-3c) revealed multifragmented implant and fixation failure. Blood tests (C-reactive protein, erythrocyte sedimentation rate, and white blood cells count), in order to rule out infection, were all within normal limits.

**Figure 4:**
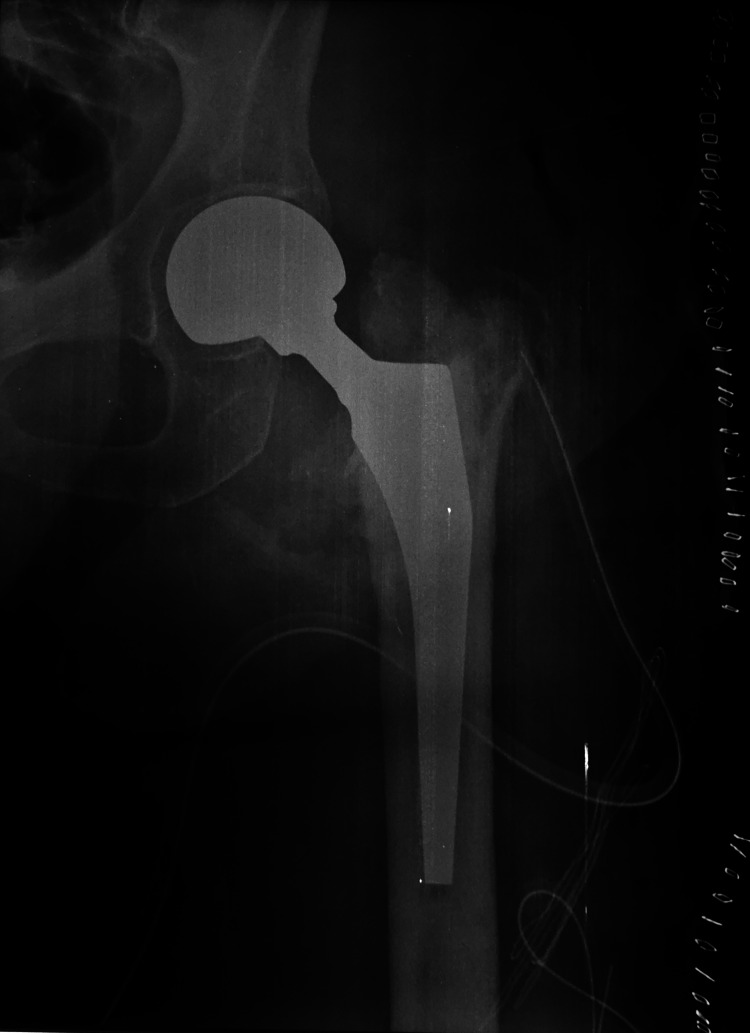
Cemented left hip hemiarthroplasty.

**Figure 5:**
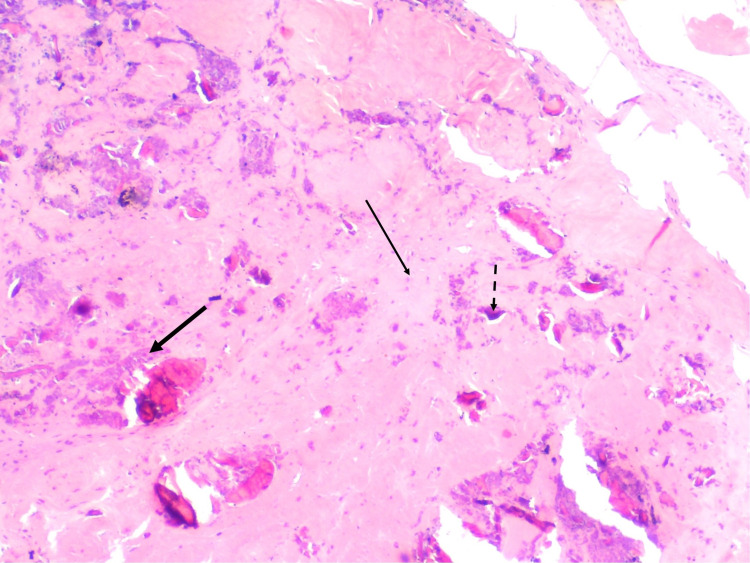
Fibrous matrix (thin arrow), bone debris (thick arrow), calcification (dashed arrow). H&E stain (x100).

**Figure 6:**
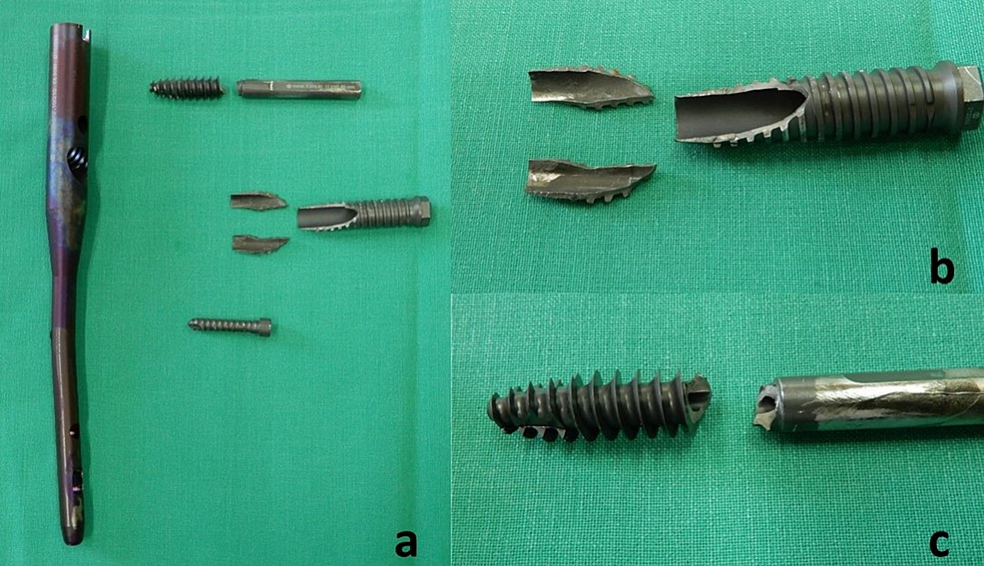
Broken implant (a), broken sleeve detail (b), and broken hip screw detail (c).

**Figure 7:**
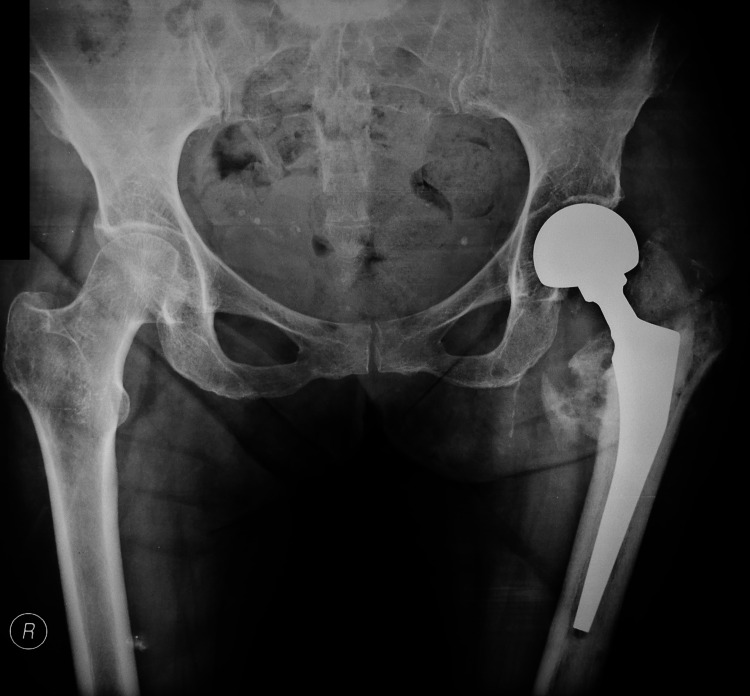
Anteroposterior pelvis x-ray, one month postoperatively.

On post-admission day 4, a revision operation took place. With the patient in the lateral decubitus position, an extended Hardinge approach was performed. Broken implant was removed and a left hip cemented hemiarhroplasty was done (Figure 4). Cultures of tissue samples taken intraoperatively were negative. The pathology exam reported calcified tissue debris and fibrosis (Figure 5). Broken implant (Figures 6a-6c) was sent to mechanical engineering laboratory for fractographic analysis. The patient came back for new radiologic evaluation one month postoperatively (Figure 7) and was again lost to follow-up thereafter.

Fractographic assessment

The status of the broken implant that was removed from the patient was assessed via fractography in order to determine the fracture mechanism that led to the catastrophic failure of the KFN assembly. As it can be seen in Figure 6, both the hip screw and the hip screw sleeve failed catastrophically. A first visual macroscopic examination of the debris revealed rough fractured surfaces of uniform texture. There was not any sign of neck formation, which would indicate plastic deformation, and the resulting fracture faces matched almost perfectly. Most of the material prior to fracture was recovered; however, some very small pieces were missing further pointing to a brittle (sudden) failure.

**Figure 8:**
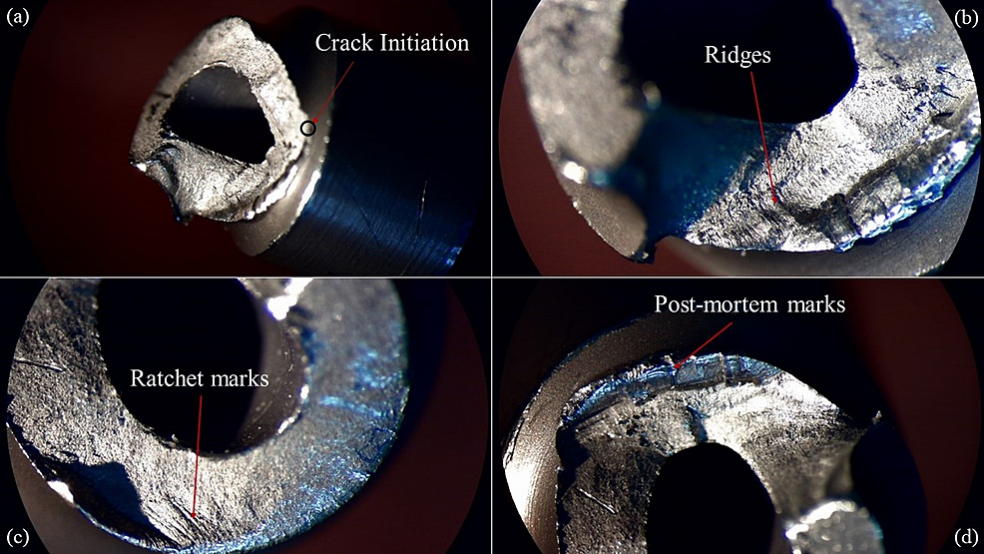
Photographs (a-d) of the broken hip screw fracture surface in 30x magnification (stereoscope).

After preliminary visual examination of the fracture surfaces, a comprehensive fractographic analysis followed using a stereoscope (Leica MZ6, Leica Microsystems, Germany). The fracture surface of the broken hip screw (Figures 8a-8d) was selected for the analysis. In the same figure, representative photographs of the fracture surface in 30x magnification are presented. A thorough examination of these images was expected to reveal the cause of the fracture in detail and provide evidence regarding the cause of failure (either overloading or fatigue).

Figure 8a presents an overview of the fracture surface, which is apparently not perpendicular to the axis of the hip screw. The crack initiation point was located near the start of the thread, where a change in diameter was present and therefore stress concentration was expected to occur. The crack propagated at an inclination of 50-55 degrees in respect to the screw axis until it reached the first thread, from where it followed the thread helix until the screw failed completely. Given the nature of the loads that the screw should withstand to retain the hip and after examining the characteristics of the crack's initial direction and propagation, the conclusion was drawn that the screw failed under combined bending and torsional loading.

Figures 8b, 8c present some characteristic types of fracture lines that they were found on the examined fracture surfaces. Ratchet marks and ridges mainly indicate step-like progression of the crack and occur parallel to the direction of crack growth [17,18]. Although they are common in both brittle and fatigue fractures, in the latter they coexist in most cases with beach marks, which appear as coaxial arcs centered at the crack initiation point and form during the stable crack growth phase. In the examined fracture surface no beach marks can be identified. The only visible marks on the surface are indicated in Figure 8d and cannot be attributed to fatigue loading since the surface locally is considerably rough and the crack initiation point is located near their left end. They are most probably post-mortem (i.e., occurred after failure) marks created from localized asperity contact of the failed surfaces in the body of the patient in the period between screw failure and the KFN debris removal operation. Furthermore, the absence of any sign of chemical action on the failed surfaces (i.e., calcification, corrosion, oxidation) strengthens the hypothesis of sudden failure due to overstressing.

In conclusion, the fracture was caused due to the combined action of bending and torsion. The absence of beach marks and the uniform roughness of the fracture surface combined with the absence of chemical attack exclude the possibility of fatigue failure. In addition, the absence of plastic deformation features, such as shear leaps, and the fact that the fracture surfaces match almost perfectly exclude the possibility of ductile fracture. Consequently, based on the above fractographic analysis, the failure of the screw is attributed to an abrupt brittle fracture due to overloading.

## Discussion

A fracture union is a race between the bony union and implant failure [11]. Several mechanisms leading to implant failure have been already described in the literature. These mechanisms can be implanted, patient or surgeon dependent, or combinations of the above. Many authors suggest that the initial fracture pattern (unstable, subtrochanteric extension, pathological fracture), poor fracture reduction during index surgery, and delayed union/nonunion are risk factors for implant failure [6,10,11,14,15].

In their 10-year retrospective review Johnson et al. [6], described 22 broken cephalomedullary nails. The site of nail failure was either at the lag screw aperture in the barrel or distal barrel taper. Based on available relevant literature [6,10-15] these are the two common sites of implant failure since these are the weakest sites from a biomechanical point of view. In their biomechanical studies, von Rüden et al. confirmed these findings showing the critical “red zone” around the insertion hole for the lag screw due to forces more than 1,800 N [13]. However, this was not the case in our study. To the best of our knowledge, this is the first case described, of a cephalomedullary nail hip screw breakage simultaneously with the corresponding sleeve. We assume that this specific nail design incorporating a hip screw sleeve that fastens in the nail transfers the load to the hip screw itself rendering this site more susceptible to fatigue or sudden load failure.

Damage during implant insertion is also a potential cause of mechanical failure. In this regard, von Rüden et al. described an implant breakage due to incorrect drilling of the insertion hole for the lag screw in one case of Proximal Femoral Nail Antirotation breakage (PFNA; Synthes, Oberdorf, Switzerland) [13]. Malalignment of the aiming device for the proximal screw or blade reamer may cause intraoperative damage to the proximal aperture in the nail, thereby predisposing the nail to failure [14]. Rappold et al. described two cases of PFN breakage. In both cases, significant metal abrasion was seen in the region of the screw hole at the site of nail breakage. This was attributed to tilting of the femoral neck screw which probably had occurred during screw insertion. They assumed that inadequate dimensioning of the guidewire which, in the presence of sclerotic bone structure, deflects cranially, ended in malposition in the screw hole. However, the authors conclude that convergent tilting of the femoral neck screw is probably of minor importance regarding the development and occurrence of nail breakage [15].

After scrutinizing our immediate postoperative x-rays, one could argue that there was a slight malreduction on the lateral view (Figure 2b). However, the tip to apex distance (TAD) was within acceptable limits (Figures 2a, 2b). After discharge, the patient was lost to follow-up and presented only nine months later with the fixation failure. The patient's compliance with postoperative instructions was questionable. Moreover, her mental status did not allow her to clearly describe or recall any preexisting pain or injury that caused the new incident. The selection of hemiarthroplasty versus total hip arthroplasty in this 50-year-old patient was decided, taking into account the patient's mental and functional status as well as her poor compliance. A hemiarthroplasty was considered to be a more stable [17] and viable solution.

After evaluating the fixation failure and patient status, questions regarding possible fracture nonunion, implant fatigue failure, potential implant defect, or a new high load incident, e.g., fall from height, arose and needed to be answered. We decided to proceed with a thorough biomechanical analysis. Fractography is a validated method for describing the cause of mechanical failure [18-20]. A scanning electron microscope could be a useful adjunct in our study but was not available. The analysis documented an abrupt brittle fracture due to sudden overloading as the cause of mechanical failure. Thus, our major concern of implant manufacturing defect or damage during implantation was ruled out.

Complications, like the one we described, may prove devastating for the patient, since implant removal is technically demanding and definitive treatment requires complex reconstruction procedures. Meticulous surgical technique and respect for implantation instructions for each specific material minimize the possibility of failures. These are, anyway, the main factors that are surgeon-controlled during surgery. Optimal reduction, correct entry point for the intramedullary nail, and proper hip screw position on both planes (anteroposterior and lateral) should be the cornerstones for such procedures. Patient health status and compliance or potential material defects are other factors that intervene during treatment but may not be fully controllable.

Material mechanical fatigue or direct high load impact may lead to nail or screw breakage. Understanding the biomechanical properties of the material planned to use is necessary. Studying the mechanism which led to failure, may help to prevent or minimize such catastrophes in the future. Moreover, this evaluation triggers the design of new or improvement of the existing implants to withstand greater forces and loads [20].

## Conclusions

Proper implant selection is critical and should be done on an individualized patient and fracture pattern basis. Poor surgical technique, implant-related issues, delayed fracture union, and poor patient compliance and health status alone or in combination can lead to breakage of the implants requiring challenging treatment options. Prevention of such catastrophic complications is crucial for the patient’s health and quality of life. Biomechanical study of the broken implant may provide useful information regarding failure causes and guide future treatment. Surgeons and mechanics should work hand in hand for implants evolution in order to optimize patient treatment.
